# Supplementary material for: Second-Trimester Dilation and Evacuation: A Simulation-Based Team Training Curriculum
Source: MedEdPORTAL. 2023 Aug 15;19:11336. doi: 10.15766/mep_2374-8265.11336 (PMC10425577; doi:10.15766/mep_2374-8265.11336)
Supplement: Supplementary file 1 — Simulation Case.docxSimulation Images.docxCritical Action Checklist.docxCase Stimuli.docxPre- and Postsimulation Learner Evaluation.docxDebriefing Guide.docxFocus Group Discussion Guide.docx [file mep_2374-8265.11336-s001.zip › B. Simulation Images.docx]

**Appendix B. Simulation Images**

**Simulation Room #1**

Author owned.

**
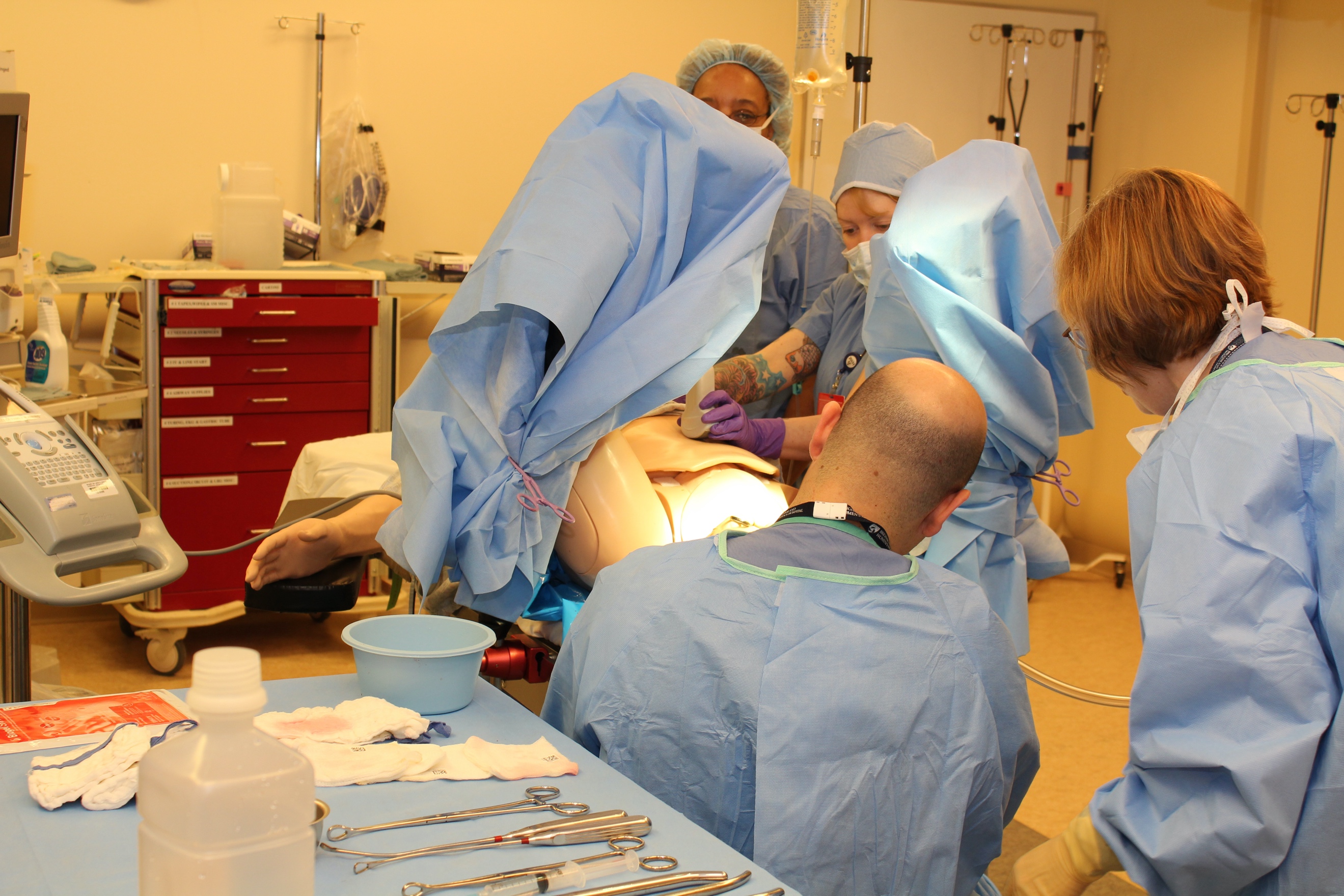
**

**Simulation Room #2**

Author owned.


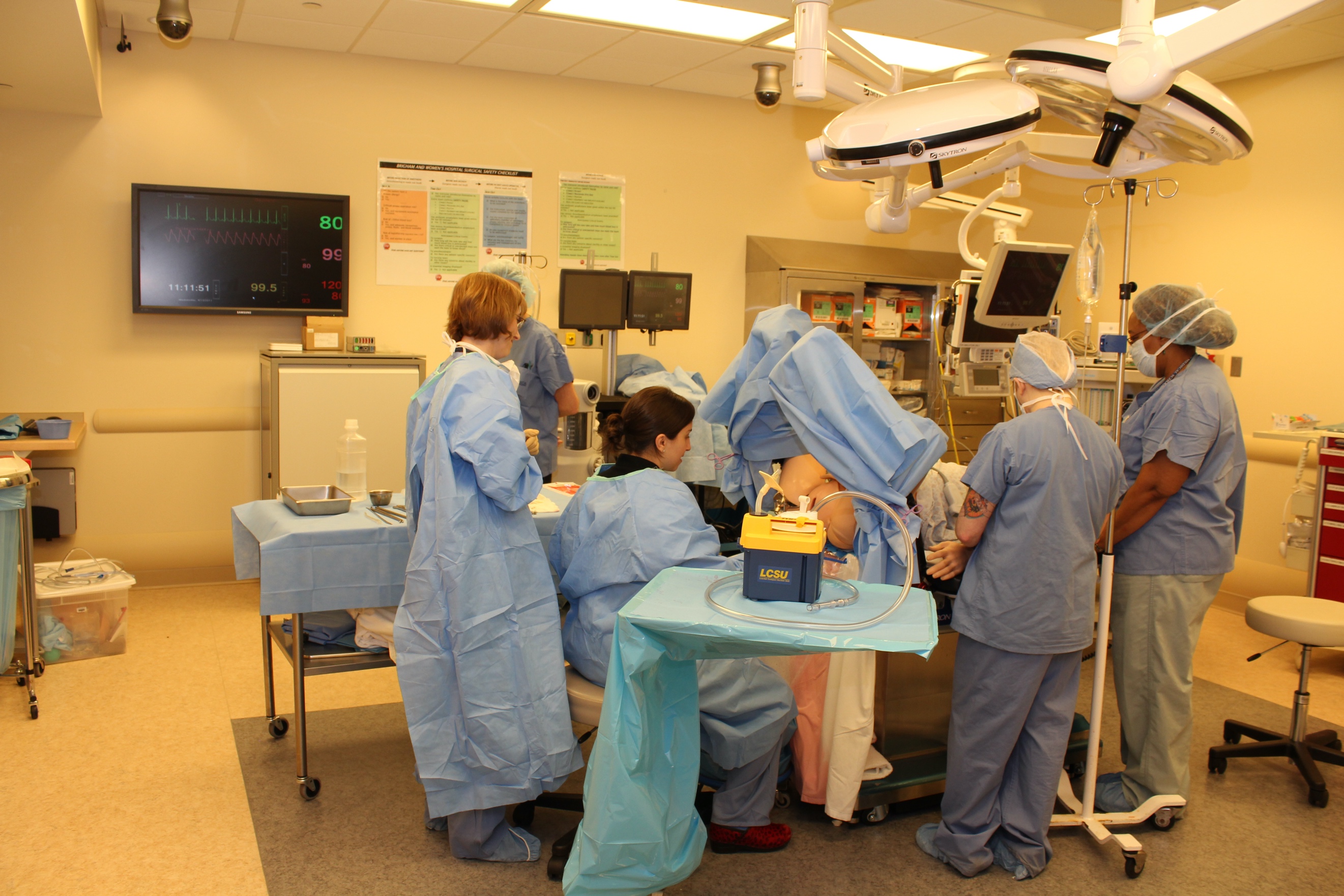


**Simulation Room #3**

Author owned.

**
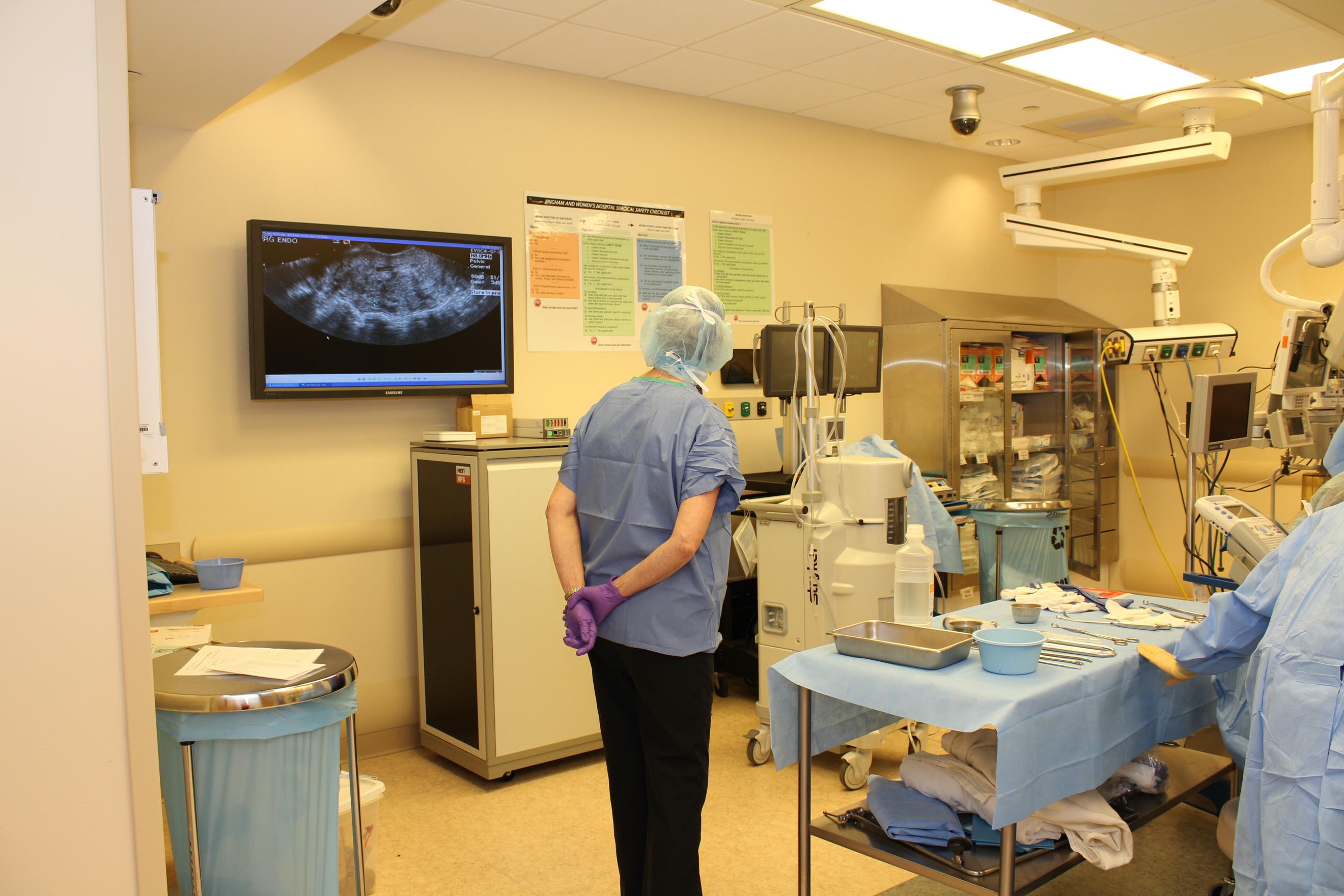
**

**View from Control Room**

Author owned.


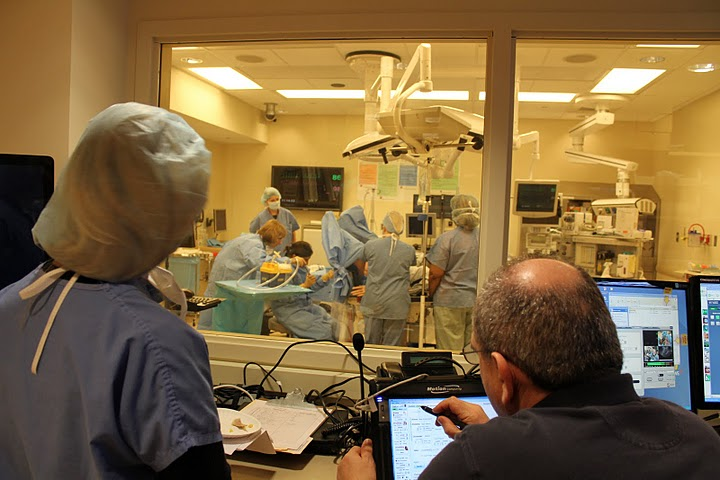


**Simulation Mannequin**

Author owned.


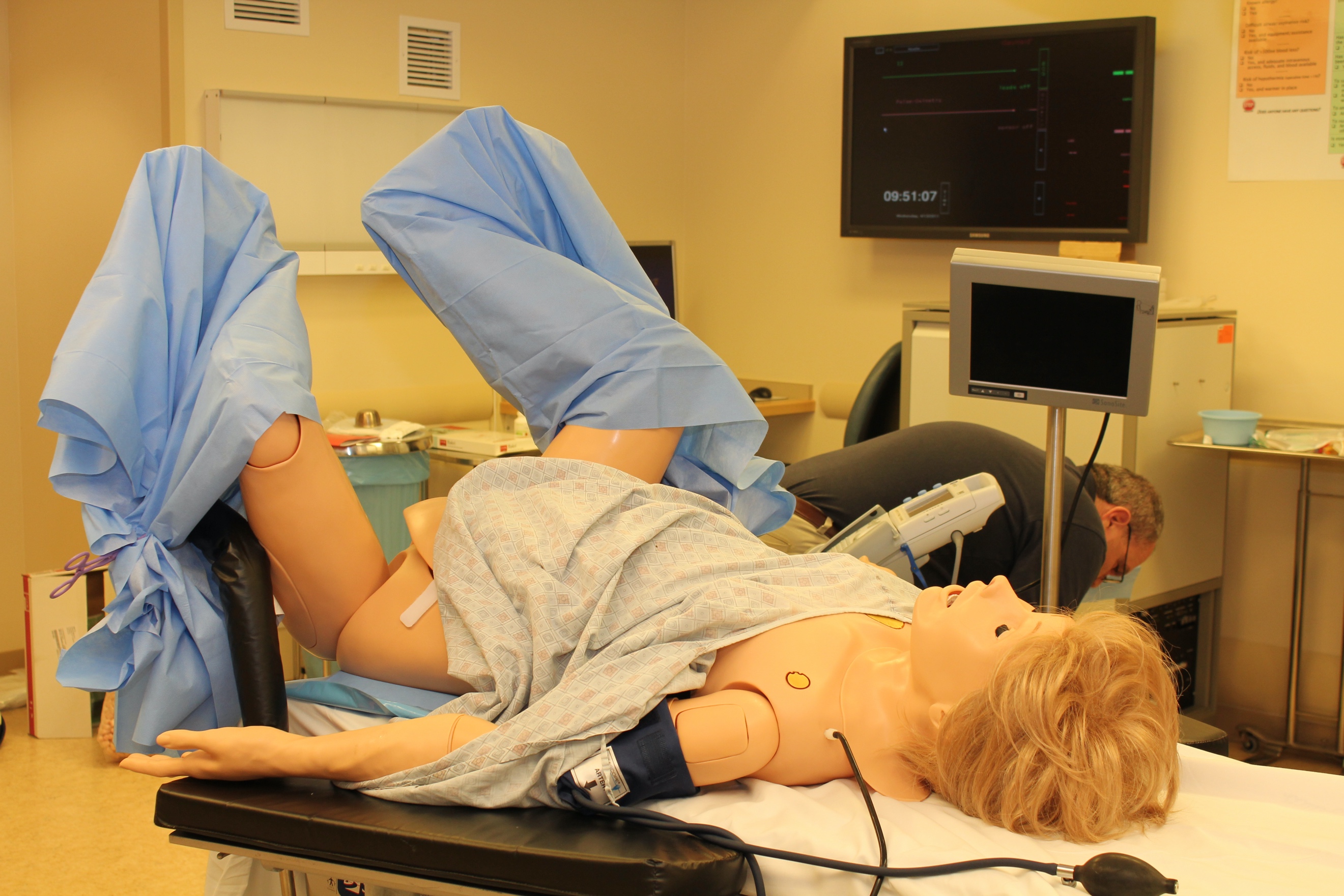


**OB/GYN Faculty Observer**

Author owned.

**
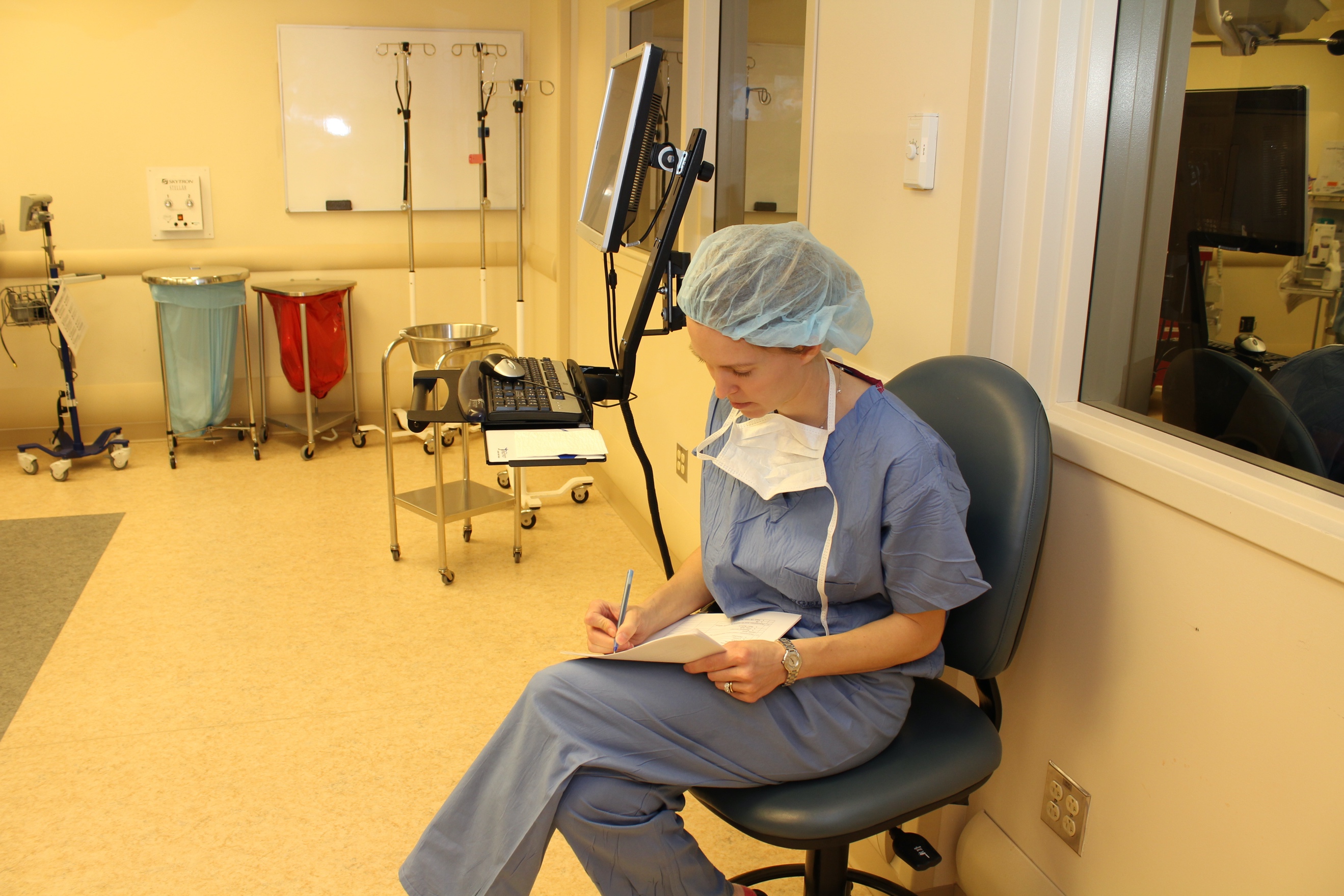
**

**Styrofoam Uterus with Packing Peanuts**

Author owned.

**
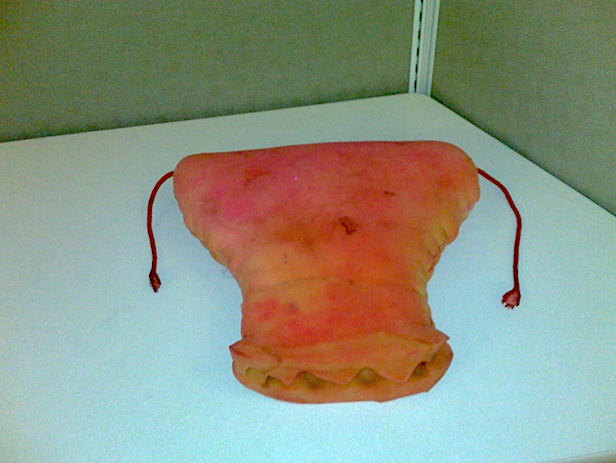
**
