# Supplementary material for: Second-Trimester Dilation and Evacuation: A Simulation-Based Team Training Curriculum
Source: MedEdPORTAL. 2023 Aug 15;19:11336. doi: 10.15766/mep_2374-8265.11336 (PMC10425577; doi:10.15766/mep_2374-8265.11336)
Supplement: Supplementary file 1 — Simulation Case.docxSimulation Images.docxCritical Action Checklist.docxCase Stimuli.docxPre- and Postsimulation Learner Evaluation.docxDebriefing Guide.docxFocus Group Discussion Guide.docx [file mep_2374-8265.11336-s001.zip › C. Critical Action Checklist.docx]

**Appendix C. Critical Action Checklist**

**D&E Hemorrhage STRATUS Simulation**

|  |  | PERFORMED | NOT PERFORMED |
| --- | --- | --- | --- |
| 1 | Surgical pause, including appropriate antibiotic coverage |  |  |
| 2 | Check with anesthesia to confirm appropriate level of sedation before starting |  |  |
| 3 | Confirms need for cervical block + vasopressin |  |  |
| 4 | Overall, D&E simulated |  |  |
|  | 1. Learner prepares syringe with 20cc 1% nesacaine + 5U vasopressin. |  |  |
|  | 1. Learner places sterile speculum and visualizes the cervix. |  |  |
|  | 1. Learner preps the cervix with betadine |  |  |
|  | 1. Learner administers 3-5cc at the 12 o’clock position of the cervix. |  |  |
|  | 1. Learner places tenaculum at 12 o’clock position of the cervix. |  |  |
|  | 1. Learner completes paracervical block with remaining 15-17cc at the 5 and 7 o’clock positions. |  |  |
|  | 1. Learner evacuates amniotic fluid with 16mm canula. |  |  |
|  | 1. Learner evacuates pregnancy using Bierer forceps |  |  |
|  | 1. Learner does final pass with suction canula to ensure completion |  |  |
|  | 1. Learner reports uterus feels empty due to ‘gritty’ sensation. |  |  |
| 5 | Demonstrates complete DDx of hemorrhage |  |  |
|  | - Atony |  |  |
|  | - Retained POCs |  |  |
|  | - Perforation |  |  |
|  | - Cervical/Vaginal laceration |  |  |
|  | - DIC |  |  |
| 6 | Communicates bleeding/atony to anesthesia |  |  |
| 7 | Requests additional help (nursing) |  |  |
| 8 | Demonstrates ability to update nurse |  |  |
| 9 | Demonstrates/communicates need for fluid resuscitation/blood products to anesthesia & staff |  |  |
| 10 | Demonstrates knowledge of medical management of atony |  |  |
|  | - Methergine |  |  |
|  | - Pitocin |  |  |
|  | - Misoprostol |  |  |
|  | - Tranexamic acid |  |  |
|  | - Carboprost tromethamine |  |  |
| 11 | Demonstrates conservative interventions for uterine atony |  |  |
|  | - Bimanual massage |  |  |
|  | - Uterine tamponade with Cook vs. Bakri vs. Foley balloon |  |  |
| 12 | Demonstrates need for transfer & transfer options |  |  |
|  | - IR |  |  |
|  | - Laparotomy tray on L&D OR Suite or transfer to Main OR for open laparotomy) |  |  |
| 13 | Demonstrates teamwork |  |  |
| 14 | Demonstrates communication skills |  |  |
