# Supplementary material for: Second-Trimester Dilation and Evacuation: A Simulation-Based Team Training Curriculum
Source: MedEdPORTAL. 2023 Aug 15;19:11336. doi: 10.15766/mep_2374-8265.11336 (PMC10425577; doi:10.15766/mep_2374-8265.11336)

**Appendix D. Case Stimuli**

**Case Scenario**

**Case Scenario**

Rebecca Ryan is a 29 yo G2P1001 who was diagnosed yesterday at 18w3d GA with an IUFD. She has been talking with her husband and her obstetrician about her delivery options including expectant management, induction of labor, and D&E. She is obese with a h/o mild asthma. She has had an SVD without complications 2 years ago. This morning she presented to the ED with a temperature of 101.3°F oral, a WBC of 19.6, normal coagulation studies, and uterine cramping. Her cramping has resulted in 2 cm of cervical dilation. Due to the evidence of uterine infection, you and your ward attending have decided to proceed with IV antibiotics and an urgent D&E in the labor and delivery operative suite.

**Ultrasound Images**

**Ultrasound Image #1**

Image by rshannonsmith, retrieved from[: https://www.flickr.com/photos/96261818@N00/382062554](https://www.flickr.com/photos/96261818@N00/382062554) on June 11, 2022. Creative Commons License associated: https://creativecommons.org/licenses/by-nc-sa/2.0/?ref=openverse.


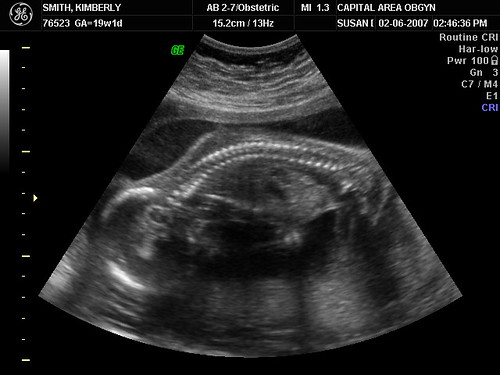


**Ultrasound Image #2**

Image by Mikael Häggström, retrieved from: <https://commons.wikimedia.org/wiki/File:Triple-line_endometrium.jpg> on June 11, 2022. Image is in the public domain.


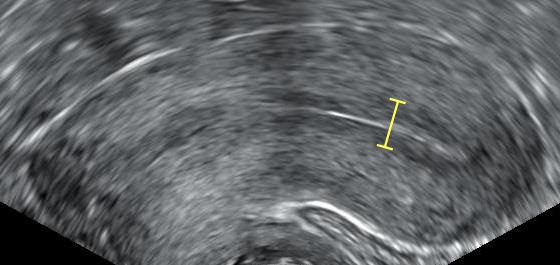


**Ultrasound Image #3**

Image by Mikael Häggström, retrieved from: <https://commons.wikimedia.org/wiki/File:Hematometra_-_postpartum.jpg> on June 11, 2022. Image is in the public domain.


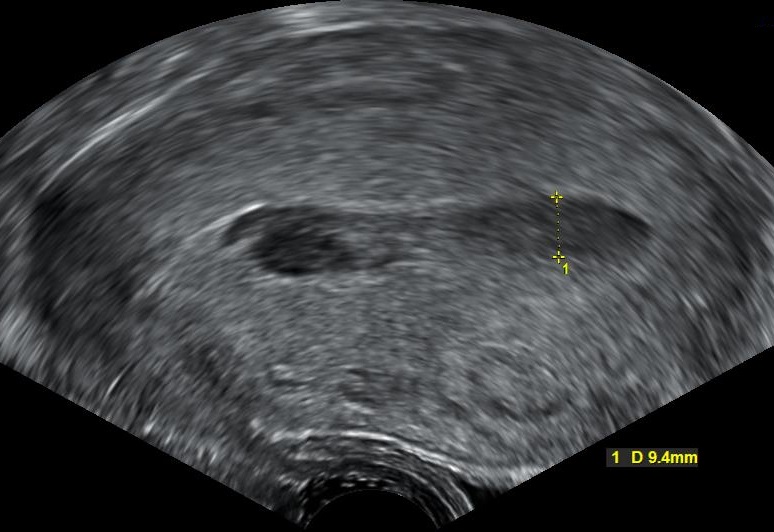

Supplement: Supplementary file 1 — Simulation Case.docxSimulation Images.docxCritical Action Checklist.docxCase Stimuli.docxPre- and Postsimulation Learner Evaluation.docxDebriefing Guide.docxFocus Group Discussion Guide.docx [file mep_2374-8265.11336-s001.zip › D. Case Stimuli.docx]
