# Supplementary material for: Second-Trimester Dilation and Evacuation: A Simulation-Based Team Training Curriculum
Source: MedEdPORTAL. 2023 Aug 15;19:11336. doi: 10.15766/mep_2374-8265.11336 (PMC10425577; doi:10.15766/mep_2374-8265.11336)
Supplement: Supplementary file 1 — Simulation Case.docxSimulation Images.docxCritical Action Checklist.docxCase Stimuli.docxPre- and Postsimulation Learner Evaluation.docxDebriefing Guide.docxFocus Group Discussion Guide.docx [file mep_2374-8265.11336-s001.zip › E. Pre- and Postsimulation Learner Evaluation.docx]

**Appendix E. Pre- and Postsimulation Learner Evaluation**

**Unique identifier (name or anonymous code word to pair pre- and postsimulation evaluation): _____________**

**Circle your year:** PGY1 PGY2 PGY3 PGY4

**Please estimate the number of procedures you have performed:**

1^st^ trimester D&Es ________

2^nd^ trimester D&Es ________

Emergent or urgent D&Es ________

**How do you feel about the following**:

| 1.) My surgical skills are: | poor | fair | average | good | excellent |
| --- | --- | --- | --- | --- | --- |
| 2.) I feel confident doing a D&E in pregnancy if necessary: | poor | fair | average | good | excellent |
| 3.) My confidence level in handling surgical variance in vaginal surgery is: | poor | fair | average | good | excellent |
| 4.) My confidence level in handling surgical emergencies in vaginal surgery is: | poor | fair | average | good | excellent |

**How do you feel about your success with various methods of learning:**

| 1.) Didactic lectures: | poor | fair | average | good | excellent |
| --- | --- | --- | --- | --- | --- |
| 2.) Simulation workshops/OSCE: | poor | fair | average | good | excellent |
| 3.) Learning by clinical experience | poor | fair | average | good | excellent |
| 4.) A combination of the above: | poor | fair | average | good | excellent |

1. **During a suction D&C, the surgeons note a midline perforation. If the uterus is not completely evacuated, all of the following options may be indicated except:**

a) complete procedure before doing anything else

b) exploratory laparotomy

c) ultrasound

d) diagnostic laparoscopy

1. **A patient has lower abdominal pain one hour after an uncomplicated elective abortion for an 8-week gestation. The uterus is tender, boggy and 12-weeks' size. There is no rebound tenderness. The most appropriate next step in the management of this patient is:**

a) Parenteral administration of Methyergonovine (Methergine)

b) Parenteral administration of Gentamicin and Clindamycin

c) Repeat aspiration

d) Laparoscopy

e) Laparotomy

1. **Which of the following steps has been shown to decrease risk from D&E:**

a) Use of intraoperative ultrasonography

b) Administration of a fetocidal agent prior to the D&E

c) Administration of a uterotonic during the D&E

d) Placement of osmotic dilators to dilate the cervix for one or more days prior to

a second trimester D&E instead of dilating only on the day of the procedure

e) Asking an assistant to keep constant fundal pressure throughout the procedure

1. **Which of the following is/are true regarding steps before first-trimester surgical abortion:**

a) It is critical to maintain a completely sterile field during the procedure.

b) Misoprostol has been shown to decrease blood loss and improve dilation prior to the procedure at 9-13 weeks gestation.

c) It is necessary to sound the uterus prior to dilation and introduction of a

canula.

d) It is necessary to do a bimanual examination prior to the procedure.

1. **Which of the following steps has been shown to decrease risk from D&E:**

a) Keeping the forceps in the lower uterine segment if possible

b) Keeping track of pregnancy elements as they are removed

c) Minimizing the number of osmotic dilators, and doing the majority of cervical

dilation on the day of the procedure

d) Use of vasopressin in the paracervical block

1. **Which of the following does not increase the risk of failed or incomplete abortion?**

a) severe uterine flexion

b) uterine anomalies

c) gestational age in the 2nd trimester

d) use of manual uterine aspiration

e) inexperienced surgeon

1. **What is the most frequent site of myometrial perforation?**

a) lower uterine segment

b) lateral midline surface

c) anterior and posterior midline surface of the active segment

d) lateral fundus

1. **Which of the following can be a treatment of atony?**

a) Manual uterine compression

b) 15-methyl PGF2alpha given as a dose of 250 micrograms

c) Methylergonovine given as a dose of 0.2 mg

d) Intravenous oxytocin

e) All of the above

**Pre- and Post-Simulation Learner Evaluation [ANSWER KEY]**

1. A
2. C
3. A
4. A
5. D
6. D
7. C
8. E
