# Supplementary material for: Second-Trimester Dilation and Evacuation: A Simulation-Based Team Training Curriculum
Source: MedEdPORTAL. 2023 Aug 15;19:11336. doi: 10.15766/mep_2374-8265.11336 (PMC10425577; doi:10.15766/mep_2374-8265.11336)
Supplement: Supplementary file 1 — Simulation Case.docxSimulation Images.docxCritical Action Checklist.docxCase Stimuli.docxPre- and Postsimulation Learner Evaluation.docxDebriefing Guide.docxFocus Group Discussion Guide.docx [file mep_2374-8265.11336-s001.zip › A. Simulation Case.docx]

| **Appendix A: Simulation Case**  **SIMULATION CASE TITLE:** Second-Trimester Dilation and Evacuation: A Simulation-based Team Training Curriculum  **AUTHORS:** Leah N. Schwartz, Andrea Pelletier, Alisa B. Goldberg, Kari Braaten, Brian Donnenfeld, Jennifer Muller, Persephone Giannarikas, Nancy Falconer, Deborah Campbell, Deborah Bartz  **LEARNER AUDIENCE:** OB/GYN Residents, all years | |
| --- | --- |
| **PATIENT NAME:** Rebecca Ryan  **PATIENT AGE:** 29 years old  **CHIEF COMPLAINT:** IUFD requiring second-trimester D&E, complicated by immediate postprocedural hemorrhage  **PHYSICAL SETTING:** Labor and delivery operative suite | |
|  | |
| **Brief narrative description of case** | The patient is a 29 yo G2P1001, diagnosed yesterday at 18w3d GA with intrauterine fetal demise (IUFD). She presented to the ED this morning with a fever and elevated WBC, as well as uterine cramping resulting in 2cm of cervical dilation. The patient subsequently undergoes an urgent second-trimester D&E procedure complicated by postprocedural hemorrhage. |
| **Primary Learning Objectives** | 1. Perform a second-trimester dilation and evacuation (D&E) procedure 2. Develop a systematic approach for the evaluation and management of hemorrhage as a complication of second-trimester D&E 3. Demonstrate teamwork and communication skills in an emergency setting. |
| **Critical Actions** | 1. Perform standard second-trimester D&E 2. Identify development of brisk bleeding 3. Evaluate uterine bleeding on physical exam and ultrasound 4. Demonstrate knowledge of complete differential diagnosis for hemorrhage    1. Atony    2. Retained POCs    3. Perforation    4. Cervical/vaginal laceration    5. DIC 5. Communicate complication to anesthesia and nursing 6. Perform uterine massage 7. Administer uterotonics, recognizing contraindications 8. Request appropriate labs to be drawn 9. Place Foley, Cook, or Bakri balloon 10. Call to coordinate patient transfer to Interventional Radiology for Uterine Artery Embolization 11. Call for open laparotomy kit to perform uterine procedures such as hypogastric artery ligation, B-Lynch suture placement, or hysterectomy. |
| **Learner Preparation or Prework** | Learners should have basic proficiency in vaginal surgery in pregnancy. The standardized OB/GYN attending who is assisting and prompting the learner will adjust their level of detail in assessment versus training in the technique of second-trimester D&E and management of obstetrical hemorrhage depending on the prior experience of the learner.  Outside of the simulation room, the resident is read a sheet of paper with the following case scenario:  *“Rebecca Ryan is a 29 yo G2P1001 who was diagnosed yesterday at 18w3d GA with an IUFD. She has been talking with her husband and her obstetrician about her delivery options including expectant management, induction of labor, and D&E. She is obese with a h/o mild asthma. She has had an SVD without complications 2 years ago. This morning she presented to the BWH ED with a temperature of 101.3°F oral, a WBC of 19.6, normal coagulation studies, and uterine cramping. Her cramping has resulted in 2 cm of cervical dilation. Due to the evidence of uterine infection, you and your ward attending have decided to proceed with IV antibiotics and an urgent D&E.”* |

| Initial Presentation | | | |
| --- | --- | --- | --- |
| **Initial vital signs** | T101.3°F oral, BP 120/80, P80, RR16, Pulse Ox 100% on room air | | |
| **Overall Setting and Appearance** | Learner enters a room that looks like a labor and delivery operative suite. They will find the mannequin already draped and in the dorsal lithotomy position on the gurney, with the anesthesiologist at the head of the gurney and the OR nurse and medical assistant at either side of the mannequin. They will be told that the patient is still awake and responsive. | | |
| **Standardized Participants (and their roles in the room at case start**) | 1. Standardized OB/GYN Attending: OB/GYN attending playing regular role   *At the start of the simulation, standardized OB/GYN attending will tell the learner:*  *“You are experienced enough that I am going to mostly just observe, ask questions, and prompt as needed.” They will provide assistance as necessary, simulating the apprentice-based training that happens routinely in the real OR.*   1. Standardized Medical assistant (MA): Staff MA playing regular role   *The MA will perform the ultrasound throughout the case. They may draw attention to the change of the ultrasound images when they occur.*   1. Standardized OR nurse: Staff OR nurse playing regular role   *The OR nurse is primarily situated within the room but may leave briefly to get blood products or other supplies. She will otherwise follow direction of the physicians, such as calling interventional radiology for uterine artery embolization when needed.*   1. Standardized Anesthesiologist: Anesthesiology attending playing regular role   *The anesthesiologist will provide verbal prompts and ques to draw the learners’ attention to the decompensation of the patient as the case progresses* | | |
| **HPI** | Rebecca Ryan is a 29 yo G2P1001 who was diagnosed yesterday at 18w3d GA with an IUFD. She has been talking with her husband and her obstetrician about her delivery options including expectant management, induction of labor, and D&E. She is obese with a h/o mild asthma. She has had an SVD without complications 2 years ago. This morning she presented to the ED with a temperature of 101.3°F oral, a WBC of 19.6, normal coagulation studies, and uterine cramping. Her cramping has resulted in 2 cm of cervical dilation. Due to the evidence of uterine infection, you and your ward attending have decided to proceed with IV antibiotics and an urgent D&E in the labor and delivery operative suite. | | |
| **Past Medical/Surgical History** | **Medications** | **Allergies** | **Family History** |
| Obesity  Asthma  SVD x1, 2 years ago | None | None | None |
| **Physical Examination** | | | |
| **General** | Patient appears non-toxic, concerned, lying in dorsal lithotomy | | |
| **GU** | Normal external genitalia. The external cervical os is open, approximately 2cm dilated. Uterus is found to be 18wks in size, retroverted, mildly tender. | | |

| Instructor Notes – Changes and CASE Branch Points | | |
| --- | --- | --- |
| **Intervention / Time point** | **Change in Case** | **Additional Information** |
| Learner introduces themselves to members of the clinical team and the patient. Leads conversation with clinical team and the patient through the surgical timeout, including a conversation about pre-operative antibiotics. | Anesthesiologist administers pre-operative antibiotics, as well as IV sedation. Patient becomes unconscious. |  |
|  |  | Learner is provided with Ultrasound Image #1 (Appendix D), demonstrating a second-trimester pregnancy. |
| The learner performs a standard second-trimester D&E. The attending will monitor the steps performed and coach when necessary. The initial ultrasound image is up on a screen in the room the whole time as the ultrasound machine is a nonfunctional prop only. |  | 1. Learner prepares syringe with 20cc 1% chloroprocaine + 5U vasopressin. 2. Learner places sterile speculum and visualizes the cervix. 3. Learner preps the cervix with betadine 4. Learner administers 3-5cc at the 12 o’clock position of the cervix. 5. Learner places tenaculum at 12 o’clock position of the cervix. 6. Learner completes paracervical block with remaining 15-17cc at the 5 and 7 o’clock positions. 7. Learner evacuates amniotic fluid with 16mm canula. 8. Learner evacuates pregnancy using Bierer forceps 9. Learner does final pass with suction canula to ensure completion 10. Learner reports uterus feels empty due to ‘gritty’ sensation. |
| The standard D&E is complete. The MA and the attending draw attention to the new ultrasound image #2 presented on the screen. | The ultrasound image on the screen changes to Ultrasound Image #2 (Appendix D) of a thin uterine stripe. |  |
|  | Brisk vaginal bleeding commences.  OB/GYN Attending comments *“Oh wait, we have a bit of bleeding.”* |  |
| MA verbally alerts learner and attending to the new finding on ultrasound | Ultrasound image on screen has changed to Ultrasound Image #3 (Appendix D) demonstrating hematometra |  |
| Learner communicates new finding of brisk bleeding to the rest of the clinical team and requests fluid resuscitation from anesthesiologist. | Anesthesiologist alerts learner to changes in patient’s vital signs.  VS: 99.9°F*,* HR 110, RR20, BP 120/70 | If learner does not communicate with anesthesiologist, OB/GYN attending will prompt “Do you want to update anesthesia about your new findings?” OR “Do you want to ask anesthesia for fluid resuscitation?” |
|  | OB/GYN Attending prompts learner for a differential diagnosis of postprocedural hemorrhage and which etiology is most likely in this patient.  OB/GYN Attending prompts learner to review of management of uterine atony. | Most likely cause of patient’s hemorrhage is uterine atony.  The OB/GYN Attending must varying their response to the learner’s response. This may include asking for a systematic way to ensure a complete differential and may include asking for the most likely etiology. |
| OR Nurse asks, *“What’s going on, is there anything I can help with?”* |  | This allows the learner to move from thinking of the differential and start thinking about interventions for the bleeding. |
| Learner asks for medications, prioritizing oxytocin (20-40U IV), methergine (0.2mg IM), misoprostol (800-1000 mcg PR), tranexamic acid (1000mg IV) |  |  |
| Learner performs uterine massage and administers medications |  |  |
|  | Anesthesiologist alerts learner to changes in patient’s vital signs.  VS: HR 128, RR20, BP 100/50  Anesthesiologist asks learner if they should call for blood products and additional support.  The OB/GYN Attending quantifies blood loss as “*quite a bit, probably close to 1 liter.*” | Given the manner our model bleeds, observed blood loss may not be accurate and thus, needs verbalization by the OB/GYN Attending. |
| Learner asks for labs (CBC, PT, PTT, INR), blood products, and communicates next step intervention which likely includes mechanical tamponade with a Foley vs. Bakri vs. Cook |  | OB/GYN Attending prompts learner as needed for additional interventions (Foley vs. Bakri vs. Cook vs. transfer), prompt learner for labs that might be desired |
| OR Nurse hands the operative team the balloon, syringe, and saline |  |  |
| Learner places Foley vs. Bakri vs. Cook |  | The OB/GYN Attending may need to instruct the learner on placement if this is a new skill set. |
| Failure to control bleeding with conservative measures. | Learner is told that bleeding continues.  Anesthesiologist alerts learner to changes in patient’s vital signs.  VS: HR 140, RR22, BP 90/40  Learner is provided with lab results:  WBC: 19.6  RBC: 4.17  Hgb: 12.0  Hct: 38.0  Plt: 200  PT: 13.8  PT-INR: 1.1 |  |
| Learner asks OR Nurse to call interventional radiology for uterine artery embolization | OR Nurse alerts team that IR is preoccupied with another patient, and they cannot accept the patient.  Bleeding continues. |  |
| Learner calls for laparotomy tray for B-lynch vs hypogastric artery ligation vs. hysterectomy | Case ends, debrief begins, team cleans and sets up for next trainee |  |

**Ideal Scenario Flow**

- Learner enters the room and immediately assumes the role of primary surgeon, leading the clinical team and patient through the surgical timeout, including requesting appropriate pre-operative antibiotics.
- After the Anesthesiologist communicates that they’ve administered general anesthesia, the learner joins the OB/GYN Attending at the perineum with a complete set of operative supplies laid out on the operative table behind the resident and attending.
- The resident is shown an ultrasound image demonstrating a second-trimester pregnancy and then prompted to begin the standard D&E procedure.
- Once the learner completes the final step of the D&E procedure—passing the Bierer forceps multiple times into the uterus to bring out some packaging peanuts that were functioning as products of conception (POC) and completing a final pass with the suction canula—the OB/GYN Attending provides reassurance and alerts the learner to a new ultrasound image demonstrating an empty uterus. The learner then checks to ensure hemostasis of the cervix, at which point the mannequin will start bleeding.
- The ultrasound image is then changed to demonstrate hematometra, corroborating the finding of vaginal bleeding but not prompting an obvious diagnosis. At this point, the learner will provide the clinical team with an update on the bleeding and the Anesthesiologist will provide the learner with updated vital signs.
- The OB/GYN Attending then prompts the learner to work through potential etiologies of bleeding and management options. The OR Nurse, OB/GYN Attending, and Anesthesiologist all follow through with requests of the learner as they go through the diagnosis and management thought process, drawing labs, providing medications, calling for blood, and calling for more attending help or other services such as Interventional Radiology (IR).
- Despite the interventions that the learner performs, the mannequin continues to bleed, and the vitals continue to worsen as the patient decompensates. The OR Nurse is instructed to alert the surgeons that IR is unavailable, eventually pushing the learner down the path to call for a laparotomy kit to start abdominal procedures, such as hypogastric artery ligation or hysterectomy.
- If the learner is too quick to call for a laparotomy set up before exhausting other interventions, such as uterotonics, a Bakri, Cook or foley balloon for uterine tamponade, or IR for uterine artery embolization, then the OB/GYN Attending prompts the learner to think of the missed interventions.
- Once the learner appropriately calls for a laparotomy the scenario ends.

**Anticipated Management Mistakes**

*Provide a list of management errors or difficulties that are commonly encountered when using this simulation case.*

1. *Failure to request medications needed for antibiotic coverage of septic abortion and for the cervical block: We found that learners often compartmentalized septic abortion as different than other infections and care on L&D. This scenario is designed to demonstrate that the need for standard D&E knowledge and skills is a requirement of any and all obstetrical practices. Learners frequently considered the prophylactic antibiotic coverage that is given at the time of D&E for uncomplicated abortion. Furthermore, residents get little exposure to providing cervical blocks in the second trimester and are, therefore, unable to go through the clinical-reasoning process to choose one regimen over another in pregnancy.*
2. *Challenge remembering and prioritizing all available uterotonics: Given the rarity of second trimester hemorrhage, learners often had trouble thinking about how some uterotonics may work less well in the less gravid uterus than the range of uterotonics used in third trimester. Therefore, the OB/GYN Attending often needed to provide additional guidance while the learner discussed available pharmacological interventions.*
3. *Delay in calling for mechanical tamponade with a balloon, uterine artery embolization by IR, and laparotomy: Learners were slow to suggest interventions that had greater team burden and greater patient consequences, particularly uterine artery embolization by IR and laparotomy. Several learners had to be prompted to recognize the gravity of the situation and escalate care, with the OR Nurse, OB/GYN Attending, and Anesthesiologist all having to emphasize the acuity of the decompensating patient. Learners were clearly strained in having to make these decisions and clear relief was frequently expressed when their decisions were met with agreement and reassurance by the rest of the team to escalate care.*
