# Supplementary material for: Second-Trimester Dilation and Evacuation: A Simulation-Based Team Training Curriculum
Source: MedEdPORTAL. 2023 Aug 15;19:11336. doi: 10.15766/mep_2374-8265.11336 (PMC10425577; doi:10.15766/mep_2374-8265.11336)
Supplement: Supplementary file 1 — Simulation Case.docxSimulation Images.docxCritical Action Checklist.docxCase Stimuli.docxPre- and Postsimulation Learner Evaluation.docxDebriefing Guide.docxFocus Group Discussion Guide.docx [file mep_2374-8265.11336-s001.zip › F. Debriefing Guide.docx]

**Appendix F. Debriefing Guide**

**Debriefing Guide**

| **Key Question** | **Points to Discuss** |
| --- | --- |
| 1. How do you think the simulation went? | - Strengths - Areas for improvement - Try to get the learner to see the gap between desired performance and actual performance - Discuss what is needed to get to fill that gap and improve performance - Outstanding questions |
| 1. What were the key steps to completing the D&E? | - Divide the simulation into stages with the standard D&E as the first stage. This ensures that the de-brief is systematically divided into smaller, potentially more memorable areas for growth |
| 1. At what point did you become concerned about postprocedural hemorrhage? How did you think about the differential diagnosis of postprocedural hemorrhage in this case? | - Try to get the learner to consider the differential diagnosis systematically (often working up through anatomy eg. vaginal laceration, cervical laceration, uterine atony, retained tissue) to ensure nothing is forgotten |
| 1. How did you think about prioritizing your approaches to managing the hemorrhage? | - Again, this should be systematic, likely prioritizing based on the order of the differential diagnosis list |
| 1. How well do you think you communicated with the team? | - **Updating team members on change in patient status**    - Discuss strategies for communicating critical changes to patient status (e.g., informing all team members simultaneously once concern for hemorrhage develops, requesting updates from anesthesia regarding patient vitals, updating team members about plans for interventions so they can anticipate next steps) - **Closed-loop communication regarding next steps in management**   - Discuss importance of directing responsibility to a specific individual for carrying out a specific task and utilizing closed loop communication to ensure the task has been completed as intended (e.g., directing anesthesia to give fluid resuscitation, directing OR nurse to give uterotonics) - **Handoff & Patient Transfer**   - Discuss strategies such as the IPASS method for enhancing information exchange during transitions in care - If time permits, you can ask the learner to demonstrate how they would have communicated with IR if they had called instead of OR nurse   **This section has been adapted from the TeamSTEPPS Framework* |
| 1. What information led you to call for IR/OR for laparotomy? Do you think you called for support at the correct time? Too early/late? |  |
