# Supplementary material for: Second-Trimester Dilation and Evacuation: A Simulation-Based Team Training Curriculum
Source: MedEdPORTAL. 2023 Aug 15;19:11336. doi: 10.15766/mep_2374-8265.11336 (PMC10425577; doi:10.15766/mep_2374-8265.11336)
Supplement: Supplementary file 1 — Simulation Case.docxSimulation Images.docxCritical Action Checklist.docxCase Stimuli.docxPre- and Postsimulation Learner Evaluation.docxDebriefing Guide.docxFocus Group Discussion Guide.docx [file mep_2374-8265.11336-s001.zip › G. Focus Group Discussion Guide.docx]

**Appendix G. Focus Group Discussion Guide**

*Focus Group Discussion Guide*

*Thank you for taking the time to participate in this focus group. As I described a bit before, the goal of this focus group is to better understand your experiences facilitating and participating in the D&E simulation curriculum. We are especially interested in learning more about the impact of the simulation curriculum on your real-life interdisciplinary team function and communication.*

*As the facilitator, I will pose several questions throughout the focus group and may follow up on specific statements an individual makes. However, we hope to have an open and honest conversation about your experiences and encourage you to engage with and respond to what others in the group have to say.*

*With that, I’d like to start by asking you some questions to learn more about your experiences.*

| **Question** | **Prompts/Probes** |
| --- | --- |
| 1. Let’s begin by having each of you share a bit about your yourself, the scope of your clinical practice, as well as how long and in what capacity you’ve been involved with the D&E simulation curriculum. | What type of provider are you?  What roles did you play in the simulation? |
| 1. Can you tell me about what your experience participating in the D&E simulations has been like? | What challenges have come up?  What would you change about the simulation? |
| 1. How did participating in the simulations impact your ability to recognize and respect the roles, responsibilities, and competence of other professions in relation to your own professional role? |  |
| 1. During what parts of the simulation did interprofessional communication feel most difficult? Most smooth? |  |
| 1. During what parts of the simulation did interprofessional team function feel most difficult? Most smooth? |  |
| 1. To what extent was the simulation helpful in resolving challenges with communication or collaboration that arise in the course of real clinical care? | What about the simulation environment made this possible?  Do you feel like the simulation environment provides a space to work out conflict?  Can you think of an example of a challenge that was addressed during the simulation? |
| 1. How did participating in the D&E simulation impact how your team functions and communicates in the course of real clinical care? |  |
