# Supplementary material for: Second-Trimester Dilation and Evacuation: A Simulation-Based Team Training Curriculum
Source: MedEdPORTAL. 2023 Aug 15;19:11336. doi: 10.15766/mep_2374-8265.11336 (PMC10425577; doi:10.15766/mep_2374-8265.11336)
Supplement: Supplementary file 1 — Simulation Case.docxSimulation Images.docxCritical Action Checklist.docxCase Stimuli.docxPre- and Postsimulation Learner Evaluation.docxDebriefing Guide.docxFocus Group Discussion Guide.docx [file mep_2374-8265.11336-s001.zip › _Educational Summary Report_11336.pdf]

MedEdPORTAL® The AAMC Journal of Teaching and Learning Resources

Original Publication Open Access

Second-Trimester Dilation and Evacuation: A Simulation-Based Team Training Curriculum

Leah N. Schwartz, MD*, Andrea Pelletier, MS, MPH, Alisa B. Goldberg, MD, MPH, Kari Braaten, MD, MPH, Brian Donnenfeld, MD, Jennifer Muller, CMA, Persephone Giannarikas, CHSOS, Nancy Falconer, RN, Deborah Campbell, RN, Deborah Bartz, MD, MPH

*Corresponding author: leah_schwartz@hms.harvard.edu

Abstract

Introduction: Despite the need for providers skilled in second-trimester dilation and evacuation (D&E) procedures, there are few second-trimester abortion training opportunities for OB/GYN residents and other health care trainees. Barriers to such training include restrictive state laws and institutional policies, lack of trained faculty, and limited procedural volume. Simulation-based D&E training is, therefore, a critical tool for OB/GYN residents and other medical professionals to achieve clinical competency. **Methods:** This simulation for OB/GYN residents centers on a 29-year-old woman at 18 weeks gestation with intrauterine fetal demise, requiring learners to perform a second-trimester D&E and manage an unexpected postprocedural hemorrhage. We designed the simulation to be used with a high-fidelity mannequin. Personnel roles required for the simulation included an anesthesiologist, medical assistant, OR nurse, and two OB/GYN faculty. Learner performance was assessed using a pre- and postsimulation learner evaluation, a critical action checklist, and a focus group with simulation facilitators. **Results:** Forty-nine residents participated over an 8-year period. Learners demonstrated improved competency performing a second-trimester D&E and increased confidence managing postprocedural hemorrhage after participating in this simulation. In addition, focus group participants reported that a majority of learners demonstrated confidence and effective communication with team members while performing in a decision-making role. **Discussion:** In addition to improving learners' clinical competency and surgical confidence for second-trimester D&E procedures, this simulation serves as a valuable instrument for the standardized assessment of learners' performance, as well as an opportunity for all participants to practice teamwork and communication in a high-acuity setting.

Keywords
Abortion, Dilation & Evacuation, Intrauterine Fetal Demise, Hemorrhage, OB/GYN, Simulation

Educational Objectives

By the end of this activity, learners will be able to:

1. Perform a second-trimester dilation and evacuation (D&E) procedure.
2. Develop a systematic approach for the evaluation and management of hemorrhage as a complication of second-trimester D&E.
3. Demonstrate teamwork and communication skills in an operative and emergency setting.

Citation:
Schwartz LN, Pelletier A, Goldberg AB, et al. Second-trimester dilation and evacuation: a simulation-based team training curriculum. *MedEdPORTAL*. 2023;19:11336.
https://doi.org/10.15766/mep_2374-8265.11336

Introduction

Pregnancy termination is exceedingly common, with over 600,000 abortions performed annually in the US, 12% of which occur after 12 weeks gestation.[1,2] While the incidence of abortion in the second trimester has remained roughly constant over the last 2 decades,[2] increased barriers to abortion following the US Supreme Court's overturning of *Roe v Wade* in June 2022[3,4] will likely increase the need for second-trimester abortion care in states where abortion remains legal. Even before the fall of *Roe,* access to second-trimester abortion was extremely limited, with 72% of abortion clinics providing care only up to 12 weeks gestation.[5] Importantly, since second-trimester abortion need is increased among individuals who are Black, have less than a high school degree, and rely on financial assistance,[6] limited access perpetuates inequity and injustice.

Despite the need for providers skilled in second-trimester dilation and evacuation (D&E), there exists a profound lack of second-

Copyright © 2023 Schwartz et al. This is an open-access publication distributed under the terms of the Creative Commons Attribution license.

trimester abortion training opportunities throughout the US and the world, a situation that is only expected to become more dire following the overturning of *Roe v Wade*.[7] Multiple factors contribute to the lack of training in D&E procedures, including restrictive state laws, anti-choice institutional policies, and lack of trained faculty.[8-11] As a result, although a majority of OB/GYN residency graduates are competent in first-trimester aspiration abortions, only 22% of residency directors believe graduates are competent in D&E procedures. This is unsurprising given that D&E is a complex procedure requiring a significant volume of cases to achieve competency and that the reported median number of second-trimester D&E procedures completed by the end of residency is four.[12] Indeed, 27% of surveyed OB/GYN residency directors and simulation faculty reported that there were additional needs for D&E simulation.[13]

To address this need, we created and assessed a high-fidelity simulation designed to improve OB/GYN residents' knowledge, confidence, and competence performing a second-trimester D&E and managing postprocedural hemorrhage, the most common serious complication of D&E. Prior studies have described the use of both low-fidelity[14] and partial trainer simulation models[15,16] for second-trimester D&E training that focus on the uterine evacuation portion of the procedure and were found to improve resident technical skill. Additional D&E simulation resources are publicly available but have not yet been evaluated.[17-19] To our knowledge, there are no resources dedicated to simulation training in second-trimester D&E in *MedEdPORTAL,* with current abortion content restricted to first-trimester dilation and suction curettage (D&C).[20-22] D&E is technically more challenging than D&C, with a higher risk of complication,[23] which likely contributes to the dearth of D&E providers. Our simulation, therefore, represents an important, novel contribution to the existing literature in that its scope includes both second-trimester D&E procedures and management of hemorrhage. In addition to serving as a capstone assessment of upper-level residents, a low-stress didactic for junior-level residents, and a team-based quality and safety exercise, this simulation has been designed to provide an inclusive teaching modality for residents who opt out of second-trimester abortion provision with real patients.

Methods

Development

Our OB/GYN residency had a total of 44 residents and was a combined, integrated residency with clinical training primarily divided between Brigham and Women's Hospital and Massachusetts General Hospital, both in Boston, Massachusetts. The residents occasionally performed D&Es in their first year of residency; however, they obtained most of their D&E training experience during their 5-week family planning rotation in PGY 2. During this rotation, residents performed between five and 15 second-trimester D&Es and developed fluency in the risks, benefits, and alternatives to D&E, options for cervical preparation, and management of complications. Residents could opt out of performing D&Es for induced abortion but could not opt out of other aspects of care. The residency also had a specific simulation curriculum embedded once a month within the weekly resident didactic program. In collaboration with experts at our institution's simulation center, family planning faculty members developed a team-based simulation to assess learner performance on second-trimester D&E and management of postprocedural hemorrhage technique (Appendix A).

To ensure that all residents could benefit from this educational module regardless of their stance on abortion, we deliberately developed the clinical case as an intrauterine fetal demise (IUFD) to remove the issue of moral objection to abortion held by some opt-out residents and to expand the generalizability of the case beyond the setting of abortion. In fact, by having IUFD as the indication and setting the simulation in labor and delivery, the simulation highlighted for learners that D&E was a pregnancy-specific procedure often utilized in routine, urgent, and emergent obstetrical settings. Similarly, we developed the case to include early sepsis to add urgency, minimize the feasibility of induction of labor as a treatment option, and underscore the fact that second-trimester D&E skills are a necessary, sometimes lifesaving prerequisite to providing full-scope maternity care. The target learners for this simulation were OB/GYN residents at all stages of training. No specific additional preparation (e.g., prereading) was required of learners or facilitators.

Environment/Equipment

We conducted this simulation at our institution's Neil and Elise Wallace STRATUS Simulation Center at Brigham and Women's Hospital.[24] We staged the center's simulated OR as an operative suite on labor and delivery (Appendix B).

The following equipment was utilized during the simulation:

- High-fidelity birthing simulator mannequin draped and placed in the dorsal lithotomy position on a surgical table: Note that most other complete or partial models could be used if there is vaginal access to a simulated cervical or uterine model.
- Second-trimester uterine model: Initially, we lacked a commercial uterine model, so we made our own using egg crate foam. To construct it, we cut two trapezoid-shaped

Copyright © 2023 Schwartz et al. This is an open-access publication distributed under the terms of the Creative Commons Attribution license.

foam pads that measured 12 inches by 10 inches by 6 inches. With the smooth sides of the foam pad placed on top of each other, we roughly sewed three of the four sides together (excluding the 6-inch side). We then flipped the foam inside out, with the textured inside representing the myometrium and the open edge representing the cervix (Appendix B). We filled this uterus with packing peanuts to simulate pregnancy tissue that could be grabbed and removed during the uterine evacuation portion of the surgery. Once we acquired an adequately sized (e.g., partum or postpartum) commercial uterus, we continued to use packing peanuts to simulate uterine contents.
- Bedside anesthesia monitor reporting heart rate, respiratory rate, blood pressure, and temperature.
- D&E surgical tray, including speculum, atraumatic tenaculum or ring forceps, Bierer forceps, kidney basin, suction tubing, and cannula.
- Suction machine.
- Ultrasound machine.
- Fake blood.
- A method of uterine tamponade such as a Bakri, Cook, or Foley (30-60 cc) balloon and syringe.
- Medications: chloroprocaine, vasopressin, methylergonovine, oxytocin, misoprostol, carboprost tromethamine, and tranexamic acid.
- Peripheral IV.
- Blood draw tubes.
- Critical action checklist (Appendix C).
- Case stimuli (Appendix D):
  - Printed case scenario.
  - Three ultrasound images to be deployed at various stages of the case: a pregnant uterus, an empty uterus with a thin endometrial stripe to indicate that the D&E was complete, and a uterus with hematometra indicating postprocedural bleeding.

Personnel
Since we ran this simulation year after year with our real clinical team, the same individuals played the same roles most every year (Appendix A). This ensured consistency across participant cohorts, especially with regard to assessment of resident learners, which was completed by the same individual in the vast majority of cases. In addition to the resident learner participant who served as the primary surgeon during the simulation, four standardized participants were utilized:

- OB/GYN attending.
- Anesthesiologist attending.
- Medical assistant.
- OR nurse.

Finally, two additional individuals were present during the simulation in supportive roles:

- OB/GYN faculty observer: responsible for observing the simulation, scoring the critical action checklist, and leading the debriefing session.
- Simulation technician: responsible for controlling the monitors and mannequin.

Implementation
This D&E simulation was one station of a 2-hour multistation simulation program on family planning techniques, with the other stations dedicated to postpartum IUD insertion, manual vacuum uterine aspiration (MVA), and hysteroscopic tubal occlusion in other rooms in the simulation center.

On the morning of the program, one OB/GYN attending and the simulation technician set up the simulated labor and delivery operating room with all the necessary equipment and familiarized the rest of the simulation team with the equipment and the case scenario. This preparation took 30 minutes.

Before participating, all learners were asked to complete the presimulation learner evaluation (Appendix E). Next, the residents, typically 12-18 annually, were divided amongst the three other stations, and one resident at a time was pulled out of the MVA training station to participate in the D&E simulation. With each D&E simulation session lasting 15-20 minutes, plus an additional 5-10 minutes for room turnover between cases, we were able to assess four to eight residents each year in D&E techniques.

Once selected to participate, the learner was read the case scenario (Appendix D) outside the simulation room by the OB/GYN faculty observer. Next, the learner was brought into the simulation room and instructed to don a gown and gloves. The OB/GYN attending told the learner that the patient was still awake and responsive and then introduced the members of the team. At that point, the learner was told that the simulation was beginning, and the OB/GYN faculty observer initiated learner evaluation with the critical action checklist (Appendix C) while sitting at a distance from the simulated OR space. Vital signs were displayed on the anesthesia cart, complete with auditory cues of pulse and oxygenation, and were controlled by the simulation technician. After the learner had gone through the steps of a complete D&E (about 5-10 minutes depending on the learner) and management of postprocedural hemorrhage (about

Copyright © 2023 Schwartz et al. This is an open-access publication distributed under the terms of the Creative Commons Attribution license.

5-10 minutes depending on the learner), the simulation ended when the learner appropriately called for a laparotomy.

Throughout the simulation, the OB/GYN attending made modifications to the questions asked, mostly as prompts, based on the learner's skill level. For those learners less proficient in D&E, the OB/GYN attending spent more time teaching and prompting. Upper-level residents who had completed the family planning rotation were held to a higher level of knowledge and technique and were asked more complicated questions regarding medication dosages and side effects to push them to and beyond the limits of their knowledge. The OB/GYN attending corrected learner responses as needed. The OB/GYN faculty observer sat on the perimeter of the simulation room or in the control room to fill out the critical action checklist (Appendix C) and did not interact with the resident.

The simulation lasted 15-20 minutes per participant, at which point the learner was brought to a separate room to debrief the case with the OB/GYN faculty observer and complete the postsimulation learner evaluation (Appendix E). The debriefing session lasted 5-10 minutes, during which time the other simulation facilitators set up the room for the next learner. In this way, the OB/GYN attending provided formative feedback, and the OB/GYN faculty observer provided summative feedback. We therefore used the simulation as a capstone assessment of the junior and senior residents before graduation.

Debriefing

Using a standardized guide (Appendix F), the OB/GYN faculty observer led a 10-minute debriefing session with each learner. The learner was first asked to summarize their perception of the case and reflect on their performance. Additional debriefing questions focused on the learning objectives of the simulation as well as on communication among team members using a modified TeamSTEPPS framework.[25] During the session, the faculty observer also reviewed the critical action checklist (Appendix C), both to reinforce the key steps of the D&E procedure and to provide the opportunity for the learner to request clarification about the simulation.

Assessment

An OB/GYN faculty observer assessed the learner's competence performing D&E procedures during the simulation using the critical action checklist (Appendix C), which was designed by OB/GYN faculty to include the actions required to competently perform a D&E procedure and manage its complications across any obstetrical setting. The checklist was scored based on whether a task was performed or not; if participants required prompting to complete a task but ultimately did so, we considered that task as performed.

All participants were asked to complete pre- and postsimulation learner evaluations (Appendix E), which were adapted from curricular materials provided by the Kenneth J. Ryan Residency Training Program in Abortion and Family Planning. The presimulation evaluation assessed number of D&E procedures performed in the past and preferred learning style, while both the pre- and postsimulation evaluations assessed confidence performing D&Es and knowledge of both first- and second-trimester abortion procedures. We calculated confidence and learning style preferences using a 5-point Likert scale (1 = *poor,* 2 = *fair,* 3 = *average,* 4 = *good,* 5 = *excellent*). We calculated knowledge scores on pre- and postsimulation evaluations, with each correct answer assigned 1 point; unanswered questions were considered incorrect. Stata version 15.0 (StataCorp) was used for analysis.

Finally, we conducted a focus group with seven simulation facilitators to assess the extent to which the simulation enabled learners to demonstrate teamwork and effective communication in a high-acuity situation, as well as the impact of the simulation on facilitators' real-life clinical team function. The project statistician used a discussion guide (Appendix G) to moderate the focus group, which was held via Zoom and lasted 1 hour. The meeting was recorded and transcribed. Two authors (Leah N. Schwartz and Andrea Pelletier) independently reviewed the transcript and met to discuss key themes identified using content analysis. Assessment of this simulation was reviewed and approved by the Partners Research Management Institutional Review Board.

Results

We facilitated the simulation once every year between 2009 and 2015 and again in 2017. During the project period, a total of 49 residents participated in the simulation. Participant level of training is described in Table 1. The median number of procedures performed prior to the simulation was assessed, with residents reporting a median of 42 first-trimester D&Cs and 10 second-trimester D&Es.

Learners showed increased knowledge after participating in the simulation (61% vs. 66%, $p = .06$), with PGY 1 and PGY 3 residents demonstrating the greatest improvement in knowledge scores. In addition, learners' mean confidence level in handling surgical emergencies in vaginal surgeries increased after participating in the simulation (2.8 vs. 3.1, $p = .001$). Confidence performing a second-trimester D&E did not change.

Copyright © 2023 Schwartz et al. This is an open-access publication distributed under the terms of the Creative Commons Attribution license.

Table 1. Residents' Year, Experience, and Learning Style Preference

Category	No. (%)	*Mdn* (IQR)
Resident year		
PGY 1	10 (20)	
PGY 2	14 (29)	
PGY 3	16 (33)	
PGY 4	9 (18)	
Total	49 (100)	
Experience		
No. of first-trimester D&Cs		42 (18-55)
No. of second-trimester D&Es		10 (3-20)
No. of urgent D&Es		5 (1-10)
No. of total procedures		60 (26-96)
Learning style[a]		
Didactic lectures		4 (3-4)
Simulation workshops/OSCE		4 (4-4)
Learning by clinical experience		5 (4-5)
Combination of the above		5 (4-5)

Abbreviations: D&C, dilation and suction curettage; D&E, dilation and evacuation; IQR, interquartile range.
[a]Rated on a 5-point Likert Scale (1 = *poor,* 5 = *excellent*).

Performance on the critical action checklist is reported in the Figure. Among all residents, the mean score was 81%. Items with the lowest completion rates included checking with anesthesia to confirm appropriate level of sedation before beginning the procedure (69%) and listing uterine perforation (28%) and cervical laceration (44%) on the differential for postabortal hemorrhage.

Finally, key themes from the focus group discussion with simulation facilitators are presented in Table 2. Thematic analysis revealed that the simulation served the dual purpose of training learners to demonstrate interprofessional teamwork in an emergent operative setting and providing the simulation facilitators with a team-based exercise to improve their interprofessional communication and collaboration.

Discussion

We created a team-based, high-fidelity simulation of second-trimester D&E and postprocedural hemorrhage for OB/GYN residents. To our knowledge, ours is the first comprehensive simulation of second-trimester D&E.

Our results highlight the overall success of this simulation as a teaching and assessment modality for learners of all levels, even those without prior experience performing D&E, as demonstrated by the fact that residents of all years, including those who had opted out of abortion training and those who had not yet completed the family planning rotation in PGY 2, achieved a mean score of 81% on the critical action checklist. The fact that PGY 1 residents scored the highest is most likely because they were prompted more often by the OB/GYN attending and the critical action checklist was scored based on completion of tasks. Moreover, although learners reported increased confidence in handling surgical emergencies in vaginal surgeries, there was no statistically significant difference in confidence performing second-trimester D&E after the simulation. We suspect that this was because residents had even less clinical experience managing postprocedural hemorrhage, making this component of the simulation more impactful on self-reported confidence.

Consistent input and investment from interdisciplinary faculty and staff throughout the development and implementation of this simulation ensured its high-fidelity nature, particularly regarding the high acuity of the scenario, which was achieved using a bleeding mannequin, auditory cues from the anesthesia monitor, and pressured prompts from facilitators. Other than updating technology when newer models became available in

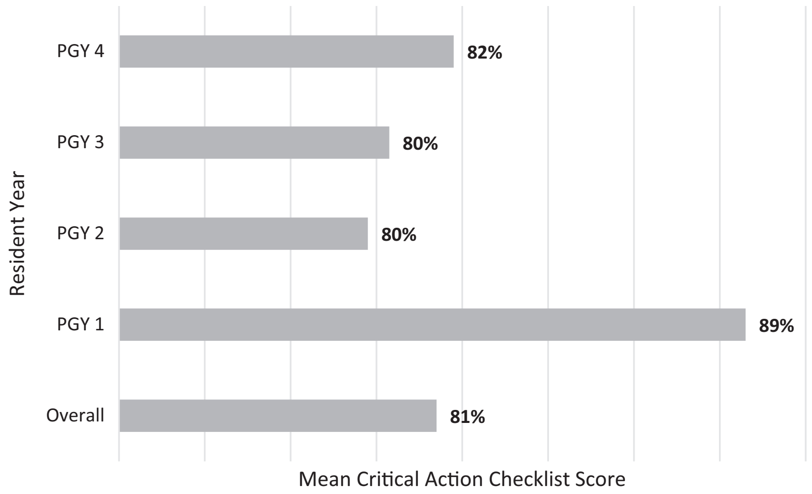

Figure. Mean score on the critical action checklist by resident year. Percentages are derived from the total number of items performed by the learner out of a maximum of 12 items on the critical action checklist (Appendix C).

Copyright © 2023 Schwartz et al. This is an open-access publication distributed under the terms of the Creative Commons Attribution license.

Table 2. Key Themes From Focus Group With Simulation Facilitators

Theme	Subtheme	Illustrative Quotes
Interprofessional teamwork	Understanding of other professional roles	"It gives you a greater appreciation of what people are thinking too and what roles they're playing.... So I think it can help you cross over all the roles in your thinking and acting." (Nurse)
	Implications for team function in clinical practice	"I don't think we realize how great the communication is, and how well we function as a team, until we take it to the simulation lab with a dummy, and we just kind of pick up where we left off when we're taking care of a real patient. And you realize how highly functional the team is because things just happen very organically." (Anesthesiologist attending)
	Effective communication	"I remember how individual residents responded and how impressed I was with their composure. Even though on the inside they felt awful, they externally demonstrated calmness and thoughtfulness and communicated well. I just remember thinking how impressed I was… they demonstrated leadership and calmness under pressure." (Anesthesiologist attending)
Preparation for clinical emergencies	Clinical decision-making	"We did the simulation, and then I think later that week, we had an emergency and it felt so much smoother because we'd just kind of gone through all the steps and the flow and what we were going to do like five times." (OB/GYN attending)
	Clinician confidence	"I think we all have a general sense of the confidence level of the residents both before and after… one in particular who I thought of as somebody who wasn't comfortable speaking out when things weren't going well, and how well she performed during the simulation. You could just see in her eyes how much confidence she felt, like she was really rising to the challenge. I think that can change the trajectory of somebody's career." (Anesthesiologist attending)
	Knowledge sharing	"Because there's multiple of us that carry the same role, I find that it is confidence boosting and reassuring to see a colleague in the same role.... It does translate then into greater confidence for each of us when we're alone in a room probably with a real patient." (OB/GYN attending)

our simulation center, we have not made targeted changes to the scenario over time. Ultimately, we would like to develop other periprocedural complication scenarios, such as amniotic fluid embolism. Since each learner completes this scenario only once, variability in the case may not be appreciated by the learner, and we very deliberately picked the hemorrhage scenario given its frequency as the most common complication from D&E. However, variability in cases would likely help the team training aspect of this exercise for our clinic staff who facilitate the simulation.

Limitations of this intervention include that the assessment instrument relied on learner self-report and that it was implemented in a heavily resourced learning environment and may not be generalizable to other contexts. We do believe, however, that this simulation could be successfully implemented in settings having fewer resources with the following modifications: using the self-created uterine model on a less sophisticated mannequin and verbally reporting blood loss volumes, using printed ultrasound images, training faculty to perform in more than one role, and having the OB/GYN attending also serve as the faculty observer who provides feedback to learners. Furthermore, because this simulation is of the entire operative case, the resultant intervention and assessment are broad.

We believe that this simulation fills an important public health need for improved second-trimester abortion training and assessment among OB/GYN residents. Perhaps equally important, we believe that it could be used to train other health care professionals, including OB/GYN physicians who never received abortion training themselves and/or who previously worked in abortion-hostile states or health systems, as well as anesthesiologists, nurses, and medical assistants, who are also critical to the provision of second-trimester D&E. This is particularly relevant for abortion educators practicing in contexts where it may be very difficult to recruit health care professionals with prior relevant training.

Appendices

A. Simulation Case.docx

B. Simulation Images.docx

C. Critical Action Checklist.docx

D. Case Stimuli.docx

E. Pre- and Postsimulation Learner Evaluation.docx

F. Debriefing Guide.docx

G. Focus Group Discussion Guide.docx

All appendices are peer reviewed as integral parts of the Original Publication.

Copyright © 2023 Schwartz et al. This is an open-access publication distributed under the terms of the Creative Commons Attribution license.

Leah N. Schwartz, MD: First-Year Resident, Department of Obstetrics and Gynecology, Brigham and Women's Hospital/Harvard Medical School; ORCID: https://orcid.org/0000-0003-1274-7577

Andrea Pelletier, MS, MPH: Statistician, Department of Obstetrics and Gynecology, Brigham and Women's Hospital/Harvard Medical School

Alisa B. Goldberg, MD, MPH: Director, Division of Family Planning, and Fellowship Director, Complex Family Planning, Department of Obstetrics and Gynecology, Brigham and Women's Hospital/Harvard Medical School

Kari Braaten, MD, MPH: Associate Fellowship Director, Complex Family Planning, Department of Obstetrics and Gynecology, Brigham and Women's Hospital/Harvard Medical School

Brian Donnenfeld, MD: Instructor, Department of Anesthesia, Brigham and Women's Hospital/Harvard Medical School

Jennifer Muller, CMA: Medical Assistant, Department of Obstetrics and Gynecology, Brigham and Women's Hospital

Persephone Giannarikas, CHSOS: Operations Manager, Neil and Elise Wallace STRATUS Center for Medical Simulation, Brigham and Women's Hospital

Nancy Falconer, RN: Nurse (Retired), Department of Obstetrics and Gynecology, Brigham and Women's Hospital

Deborah Campbell, RN: Nurse (Retired), Department of Obstetrics and Gynecology, Brigham and Women's Hospital

Deborah Bartz, MD, MPH: Associate Clerkship Director, Obstetrics and Gynecology Clerkship, and Director, Kenneth J. Ryan Program in Abortion and Family Planning, Department of Obstetrics and Gynecology, Brigham and Women's Hospital/Harvard Medical School; ORCID: https://orcid.org/0000-0002-3096-9715

Disclosures
None to report.

Funding/Support
None to report.

Ethical Approval
The Partners Research Management Institutional Review Board approved this project.

References

1. Induced abortion in the United States: fact sheet. Guttmacher Institute. September 2019. Accessed June 16, 2023. https://www.guttmacher.org/fact-sheet/induced-abortion-united-states
2. Kortsmit K, Jatlaoui TC, Mandel MG, et al. Abortion surveillance—United States, 2018. *MMWR Surveill Summ*. 2020;69(7):1-29. https://doi.org/10.15585/mmwr.ss6907a1
3. *Roe v Wade,* 410 US 113 (1973).
4. *Dobbs v Jackson Women's Health Organization*, 14 S Ct 2228 (2022).
5. Jones RK, Ingerick M, Jerman J. Differences in abortion service delivery in hostile, middle-ground, and supportive states in 2014. *Womens Health Issues*. 2018;28(3):212-218. https://doi.org/10.1016/j.whi.2017.12.003
6. Fuentes L, Jerman J. Distance traveled to obtain clinical abortion care in the United States and reasons for clinic choice. *J Womens Health (Larchmt)*. 2019;28(12):1623-1631. https://doi.org/10.1089/jwh.2018.7496
7. Vinekar K, Karlapudi A, Nathan L, Turk JK, Rible R, Steinauer J. Projected implications of overturning *Roe v Wade* on abortion training in U.S. obstetrics and gynecology residency programs. *Obstet Gynecol*. 2022;140(2):146-149. https://doi.org/10.1097/AOG.0000000000004832
8. An Act to Create 20.9273 of the Statutes; Relating to: Prohibiting Performance of and Funding or Providing Property for Abortions and Other Services by Certain Employees and Entities, SB 260, 2021-2022 Leg (Wis 2021). March 31, 2021. Accessed June 16, 2023. https://docs.legis.wisconsin.gov/2021/proposals/reg/sen/bill/sb260
9. León N. *Diagnosing Discrimination: Barriers Facing Health Care Providers Who Support and Perform Abortion*. National Women's Law Center; 2018. Accessed June 16, 2023. https://nwlc.org/wp-content/uploads/2018/04/ProviderDiscrimReport2018.pdf
10. Zeldovich VB, Rocca CH, Langton C, Landy U, Ly ES, Freedman LR. Abortion policies in U.S. teaching hospitals: formal and informal parameters beyond the law. *Obstet Gynecol*. 2020; 135(6):1296-1305. https://doi.org/10.1097/AOG.0000000000003876
11. Turk JK, Landy U, Chien J, Steinauer JE. Sources of support for and resistance to abortion training in obstetrics and gynecology residency programs. *Am J Obstet Gynecol*. 2019;221(2): 156.E1-156.E6. https://doi.org/10.1016/j.ajog.2019.04.026
12. Steinauer JE, Turk JK, Pomerantz T, Simonson K, Learman LA, Landy U. Abortion training in US obstetrics and gynecology residency programs. *Am J Obstet Gynecol*. 2018;219(1): 86.E1-86.E6. https://doi.org/10.1016/j.ajog.2018.04.011
13. DeStephano CC, Nitsche JF, Heckman MG, Banks E, Hur HC. ACOG Simulation Working Group: a needs assessment of simulation training in OB/GYN residencies and recommendations for future research. *J Surg Educ*. 2020;77(3):661-670. https://doi.org/10.1016/j.jsurg.2019.12.002
14. Baldwin MK, Chor J, Chen BA, Edelman AB, Russo J. Comparison of 3 dilation and evacuation technical skills models. *J Grad Med Educ*. 2013;5(4):662-664. https://doi.org/10.4300/JGME-D-13-00049.1
15. York SL, Migas S, Haag J, et al. Creation and initial assessment of a second-trimester uterine model. *Simul Healthc*. 2014;9(3): 199-202. https://doi.org/10.1097/SIH.0000000000000042
16. York SL, McGaghie WC, Kiley J, Hammond C. Implementation and evaluation of a dilation and evacuation simulation training

Copyright © 2023 Schwartz et al. This is an open-access publication distributed under the terms of the Creative Commons Attribution license.

curriculum. *Contraception*. 2016;93(6):545-550. https://doi.org/10.1016/j.contraception.2016.02.002

17. Costescu D. Multiple-module simulation of dilation and evacuation facilitation guide. Innovating Education in Reproductive Health. Accessed June 16, 2023. https://www.innovating-education.org/2016/03/multiple-module-simulation-of-dilation-and-evacuation-facilitation-guide/
18. Lohr PA. Dilation and evacuation animation. Innovating Education in Reproductive Health. March 11, 2022. Accessed June 16, 2023. https://www.innovating-education.org/2022/03/dilation-and-evacuation-animation/
19. York S. *D&E Training Model*. Innovating Education in Reproductive Health; date unknown. Accessed June 16, 2023. https://www.innovating-education.org//wp-content/uploads/2013/07/2nd_York_Description_DE-Training-Model-with-silicone-cervix-York.pdf
20. Storey A, White K, Treder K, Woodhams E, Bell S, Cannon R. First-trimester abortion complications: simulation cases for OB/GYN residents in sepsis and hemorrhage. *MedEdPORTAL*. 2020;16:10995. https://doi.org/10.15766/mep_2374-8265.10995
21. Davis J, Weinberger L. Emergency medicine simulation scenario: first-trimester “septic” abortion. *MedEdPORTAL*. 2011;7:9027. https://doi.org/10.15766/mep_2374-8265.9027
22. Goodman S, McNeil S, Shih G. Simulation for managing hemorrhage as a complication of uterine aspiration. *MedEdPORTAL*. 2015;11:10296. https://doi.org/10.15766/mep_2374-8265.10296
23. Bartlett LA, Berg CJ, Shulman HB, et al. Risk factors for legal induced abortion-related mortality in the United States. *Obstet Gynecol*. 2004;103(4):729-737. https://doi.org/10.1097/01.AOG.0000116260.81570.60
24. Neil and Elise Wallace STRATUS Center for Medical Simulation. Brigham and Women’s Hospital. Accessed June 16, 2023. https://www.brighamandwomens.org/emergency-medicine/stratus-center-for-medical-simulation
25. Pocket guide: TeamSTEPPS. Agency for Healthcare Research and Quality. Accessed June 16, 2023. https://www.ahrq.gov/teamstepps/instructor/essentials/pocketguide.html

Received: June 13, 2022
Accepted: May 2, 2023
Published: August 15, 2023

Copyright © 2023 Schwartz et al. This is an open-access publication distributed under the terms of the Creative Commons Attribution license.
